# Potential mechanisms of zinc sulfate in inhibiting porcine reproductive and respiratory syndrome virus replication: attenuation of oxidative stress, inflammatory response, and apoptosis

**DOI:** 10.3389/fvets.2025.1663508

**Published:** 2025-09-04

**Authors:** Huizhen Yang, Chengzi Zhang, Qianfan Li, Zixuan Wu, Na Sun, Wei Yin, Kuohai Fan, Hongquan Li

**Affiliations:** ^1^Shanxi Key Lab. for Modernization of TCVM, College of Veterinary Medicine, Shanxi Agricultural University, Jinzhong, China; ^2^Laboratory Animal Center, Shanxi Agricultural University, Jinzhong, China

**Keywords:** zinc sulfate, porcine reproductive and respiratory syndrome virus, transcriptomic analysis, oxidative stress, inflammatory factors, apoptosis

## Abstract

**Introduction:**

This study aimed to elucidate the mechanism of zinc sulfate against porcine reproductive and respiratory syndrome virus (PRRSV) through transcriptomic data and experimental validation.

**Methods:**

Initially, the expression of PRRSV N gene and protein were quantified using qPCR and immunofluorescence, respectively. High-throughput RNA sequencing was performed to analyze global gene expression changes in PRRSV-infected Marc-145 cells treated with zinc sulfate. Transcriptomic data were subjected to bioinformatic analyses, including Venn diagram assessments, protein–protein interaction network construction using the STRING database, and identification of hub genes via Cytoscape 3.10.0. Functional enrichment analysis of Gene Ontology and KEGG pathways were performed using R (v4.4.3). Subsequently, oxidative stress parameters in PRRSV-infected Marc-145 cells treated with zinc sulfate were detected using biochemical assays. The modulatory effects of zinc sulfate on inflammatory response and apoptosis were evaluated through qPCR and western blot, measuring the expression of cytokines (IL-6, IL-8, TNF-*α*, IL-10), apoptosis-related proteins (Caspase-3, Bax, Bcl-2), and key components of the NF-κB pathway. Finally, flow cytometry was employed to assess cellular apoptosis rates.

**Results:**

The result demonstrated that zinc sulfate significantly suppressed PRRSV replication. The transcriptomic analysis revealed that compared to the PRRSV-infected group, there were 14 upregulated and 50 downregulated targets in zinc sulfate treatment group. Among these, ten core downregulated and upregulated targets were well enriched in the inflammation and apoptosis pathways, respectively. The experimental verification results demonstrated that compared to the PRRSV-infected group, zinc sulfate treatment significantly diminished intracellular reactive oxygen species (ROS) and malondialdehyde (MDA) levels, while elevating the enzymatic activities of superoxide dismutase (SOD) and catalase (CAT) (*p* < 0.05). It also suppressed the expression of IL-6, IL-8, TNF-*α*, while upregulating IL-10 (*p* < 0.05). In addition to, it also upregulated the phosphorylation levels of IκBα and p65 (*p* < 0.05), and decreased the expression of Caspase-3, cleaved-Caspase-3, Bax, while enhancing Bcl-2 (*p* < 0.05). Simultaneously, flow cytometry analysis further confirmed that zinc sulfate substantially attenuated late-stage and overall apoptosis rates in PRRSV-infected cells (*p* < 0.05).

**Conclusion:**

These results implied that zinc sulfate restricted oxidative stress, diminished inflammatory response and induced apoptotic suppression to confer protection against PRRSV infection.

## 1 Introduction

Porcine reproductive and respiratory syndrome (PRRS) is a highly contagious and economically significant disease that profoundly impacts the global swine industry. It is caused by the porcine reproductive and respiratory syndrome virus (PRRSV) and predominantly induces reproductive failure in pregnant sows and respiratory disorders in growing-finishing pigs, nursery pigs, and piglets (1). PRRSV is an enveloped, single-stranded positive-sense RNA virus classified under the Arteriviridae family, genus Arterivirus. Since 2006, the incidence of PRRSV genomic recombination has markedly increased, leading to a progressively more intricate epidemiological framework of PRRSV (2). In China (2021–2023), several circulating strains exhibited multi-lineage coexistence and diversity, with NADC30-like PRRSV-2 emerging as the predominant variant (3, 4). Notably, NADC34-like PRRSV-2 is gaining prominence as a major epidemic strain, and its prevalence is projected to expand further by 2025 (5). Current commercial PRRSV vaccines exhibitlimited efficacy against NADC34-like PRRSV-2 (6, 7). Due to the limited efficacy of current vaccines against heterologous strains, protective immunity remains constrained to closely related viral variants (8, 9). Thus, although vaccination remains the cornerstone of PRRSV control, reliance solely on existing vaccines is insufficient for complete protection or eradication, underscoring the urgent need for integrated alternative strategies.

Zinc, the second most abundant essential trace element in living organisms, plays a vital role in numerous physiological processes, including, among others, immune regulation, signal transduction, redox homeostasis, as well as cellular proliferation and apoptosis (10–12). Clinical research has evidenced zinc’s robust antiviral capabilities, as illustrated by Ghaffari et al. and Tavakoli et al., showing that zinc oxide nanoparticles effectively inhibited infections induced by influenza A virus (H1N1) and herpes simplex virus type 1 (HSV-1) *in vitro* (13, 14). Moreover, Khan et al.’s research further demonstrated that zinc supplementation effectively suppressed respiratory syncytial virus (RSV) replication *in vitro* (15). Additionally, clinical studies on viral infections, such as human immunodeficiency virus (HIV) and hepatitis C virus (HCV), have shown that zinc supplementation can significantly reduce viral load in target tissues (16). Importantly, therapeutic administration of zinc sulfate in COVID-19 patients has been shown to accelerate clinical recovery and reducing mortality (17, 18). However, there is limited research on the antiviral properties of zinc in veterinary medicine. Thus far, only the research team led by Zhang et al. at the Hubei Provincial Key Laboratory of Animal Nutrition and Feed Science has demonstrated zinc’s efficacy against porcine epidemic diarrhea virus (PEDV) infection (19). Notably, all the aforementioned viruses (H1N1, RSV, HIV, HCV, COVID-19, and PEDV) share a striking commonality: they all have RNA genomes. This implies that elevated zinc concentrations in cells or blood probably can effectively inhibit or suppress RNA viral infections.

Considering PRRSV is a single-stranded RNA virus, and based upon Guo et al.’s discovery that the zinc ionophore pyrithione elevates intracellular zinc ion concentrations to inhibit PRRSV replication (20), we hypothesized that increasing zinc ion availability could effectively suppress PRRSV replication. This hypothesis was strongly supported by our previous experimental findings demonstrating that both zinc chloride and zinc sulfate treatments significantly reduced PRRSV viral titers (21). Notably, zinc sulfate - as the predominant and economically viable zinc supplement in porcine nutrition - has additionally demonstrated clinical efficacy as a zinc supplementation therapy in COVID-19 management (17), thereby justifying its selection as the focal compound for examining PRRSV replication inhibition in our experimental design. Subsequent analyses revealed that zinc sulfate exerted significant inhibitory effects on PRRSV replication in both Marc-145 and porcine alveolar macrophage (PAM) cell lines (21). Crucially, we found that zinc sulfate exerts multifaceted antiviral activity—not merely blocking PRRSV adhesion and cellular entry but also significantly impairing intracellular replication and subsequent release (21). Nevertheless, the precise molecular mechanisms underlying zinc sulfate’s antiviral activity against PRRSV remain elusive.

In this study, PRRSV-infected Marc-145 cell model was employed to systematically investigate zinc sulfate’s anti-PRRSV mechanisms through integrated multi-omics approaches, including transcriptomics, real-time quantitative PCR, immunoblot, and flow cytometry. Mechanistically, the results revealed zinc sulfate suppressed PRRSV replication through coordinated regulation of three cellular processes: (1) oxidative stress, (2) inflammation, and (3) apoptosis. These results offer novel mechanistic insights into zinc sulfate’s antiviral action and pinpoint host cellular pathways as potential targets for PRRSV intervention strategies.

## 2 Materials and methods

### 2.1 Cell culture and PRRSV amplification

MARC-145 cells were employed as the experimental model for elucidating zinc’s anti-PRRSV mechanisms *in vitro*, as this cell line possesses well-documented susceptibility to PRRSV infection and exceptional experimental reproducibility, making it a commonly chosen *in vitro* cell model for studying the infection mechanism of PRRSV. Marc-145 cells were purchased from the China Veterinary Drug Supervision Institute (CVCC Code: CL32), cultured and preserved by Shanxi Provincial Key Laboratory for Modernization of TCVM at the College of Veterinary Medicine, Shanxi Agricultural University (Mingxian South Road, Taigu County, Shanxi Province, China). Marc-145 cells were cultured in high glucose Dulbecco’s modified Eagle’s medium (DMEM) containing 10% fetal bovine serum (FBS) and 1% penicillin and streptomycin at 37°C in 5% CO_2_ and a humidified atmosphere. Upon reaching 80–90% confluence, the Marc-145 cells were trypsinized for subsequent passage. Following quantification, cells were seeded into 25 cm^2^ cell culture flasks for subsequent propagation. Once Marc-145 cells reached 80–90% confluency again, they were eligible for subsequent experimental procedures.

The PRRSV JS-1 strain, with a 50% tissue culture infective dose (TCID50) of 10^−5^·^55^ mL^−1^, was kindly provided by the Jiangsu Academy of Agricultural Sciences. The viral stock was propagated in Marc-145 cells and subsequently cryopreserved in our laboratory. Firstly, Marc-145 cells were cultured in a high glucose DMEM medium with 10% FBS and 1% penicillin and streptomycin until reaching 80% confluence. The PRRSV stock was serially diluted (10-fold) in serum-free DMEM. Subsequently, Marc-145 cells were infected with 5 mL of the diluted PRRSV suspension and incubated at 37°C with 5% CO₂ for 2 h to allow viral adsorption. Following incubation, the virus-containing medium was removed and cell monolayers were gently washed three times with phosphate-buffered saline (PBS). Subsequently, infected cells were maintained in high-glucose DMEM containing reduced serum (2% FBS) for 60 h at 37°C with 5% CO₂. When 80–90% cytopathic effect (CPE) was observed, the medium was discarded and infected cells were harvested by scraping for further extraction of PRRSV. The collected Marc-145 cells were subjected to cycles of freezing and thawing for three times between −20°C and room temperature. Later, the cell lysate was centrifuged at 1760 × g for 30 min at 4°C to pellet the cellular debris and the supernatant was filtered through a 0.22 μm pore-size sterile membrane using a syringe filter. At last, the clarified virus solution was aliquoted into sterile cryovials and stored at −80°C for long-term preservation.

### 2.2 Cells treatment protocol and experimental groups

The PRRSV stock was diluted to 100 TCID₅₀·mL^−1^ in high-glucose DMEM supplemented with 2% FBS. The stock solution of 50 mmol·L^−1^ zinc sulfate was prepared by dissolving ZnSO₄·7H₂O in distilled water at room temperature and then, the stock solution was further diluted in high-glucose DMEM supplemented with 2% FBS to obtain final concentrations of 100, 50, and 12.5 μmol·L^−1^. Marc-145 cells were cultured in high-glucose DMEM enriched 10% FBS and 1% penicillin–streptomycin until reaching 80% confluence at 37°C in 5% CO_2_. Then, the cells were inoculated with 100 TCID₅₀·mL^−1^ PRRSV and incubated at 37°C for 2 h with 5% CO₂ to facilitate viral adsorption. After 2 h, the medium containing PRRSV was discarded and the cells were treated with maintenance medium containing 100, 50, and 12.5 μmol·L^−1^ zinc sulfate at 37°C in 5% CO_2_ for 48 h. After 48 h of incubation, Marc−145 cells were harvested. Meanwhile, a blank group was set up only with cells but without zinc sulfate and PRRSV, and the PRRSV control group was set up with cells and PRRSV but without zinc sulfate. The experiment was repeated three times individually.

### 2.3 Preparing the Marc-145 cells for transcriptomic sequencing

To elucidate the differential gene expression profiles and affected pathways associated with zinc sulfate treatment upon PRRSV infection, transcriptomic analysis was performed on Marc-145 cells from three experimental groups: non-infected control (Marc-145 cells), PRRSV-infected cells, PRRSV-infected cells treated with 100 μmol·L^−1^ zinc sulfate. Cell treatment was followed the same protocol as described in “Section 2.2.” In addition, each group included three independent replicates to ensure statistical robustness. At 48 h post-inoculation, cells were lysed in TRIzol reagent and immediately stored at −80°C to preserve RNA integrity. Total RNA extraction and subsequent RNA-seq were conducted by Sangon Biotech Co., Ltd. (Shanghai, China) using an Illumina platform. Sequencing data were analyzed for differentially expressed genes (DEGs) and enriched pathways under different treatment conditions.

### 2.4 Venn analysis, protein–protein interaction (PPI) network construction, gene ontology (GO) and KEGG analysis, and regulatory network construction

To elucidate the molecular mechanisms underlying zinc sulfate’s antiviral activity against PRRSV, we performed high-throughput transcriptome sequencing to analyze the gene expression profiles of PRRSV-infected Marc-145 cells treated with 100 μmol·L^−1^ zinc sulfate for 48 h. The sequencing data were processed and analyzed using R version 4.4.3 (R Core Team), with differential gene expression visualized through volcano plots to demonstrate the statistical significance of transcriptional alterations. In order to complement transcriptome analysis, PRRS-related target genes were retrieved from the GeneCards database.^1^ Bioinformatics-based comparative analysis was conducted on the Bioinformatics Venn platform^2^, where differentially expressed genes (both upregulated and downregulated) from zinc sulfate-treated samples were systematically compared against known PRRS-associated targets using Venn diagrams. This computational intersection approach identified core molecular targets potentially mediating zinc sulfate’s therapeutic effects against PRRSV.

The intersecting target genes obtained in Venn analysis were input into the STRING database’s Multiple Proteins module to generate a PPI network, and the resulting interaction data was exported in TSV format. Subsequently, the network file was imported into Cytoscape (version 3.10.0) for topological analysis. Using the cytoHubba plugin, we applied the maximal clique centrality (MCC) algorithm (confidence score threshold ≥40%) to systematically rank network nodes by their topological significance. Based on this quantitative assessment, the top ten highest-ranking genes were identified as potential core regulatory elements in zinc sulfate’s antiviral mechanism.

The intersecting targets obtained in Veen analysis were subjected to comprehensive functional annotation using R (version 4.4.3). Functional enrichment analysis was performed through the clusterProfiler package (version 4.12.0), with GO term enrichment (BP/CC/MF) conducted via the enrichGO function based on the *Sus scrofa* annotation database (org.Ss.eg.db). Enriched terms were ranked by statistical significance, and the top 10 most significant categories from each ontology Biological Process (BP), Cellular Component (CC), and Molecular Function (MF) were visualized as bar plots. Concurrently, KEGG pathway analysis identified the 30 most statistically significant pathways (*p* < 0.05), which were subsequently represented in an enrichment bubble plot, illustrating both pathway relevance and enrichment magnitude.

By integrating multi-omics analysis and PPI mapping, we established an interactive regulatory network that systematically delineates the interplay among zinc sulfate, core targets, and key signaling pathways. High-confidence hub genes (top 10 in betweenness centrality and degree) were selected as pivotal nodes, while KEGG pathway enrichment (*p* ≤ 0.05) provided mechanistic context. Edge weights were assigned based on interaction confidence scores (STRING database) and functional associations. The resultant network was structurally optimized for visualization using Cytoscape (version 3.9.1), with dynamic regulatory relationships depicted through directional edges (Zinc sulfate-Targets-Pathways), thereby revealing the hierarchical architecture of zinc sulfate-mediated antiviral regulation.

### 2.5 Quantitative real-time PCR (qPCR) analysis of PRRSV N gene copies and inflammation-related genes expression levels

The experiment groups included a blank control group, a PRRSV-infected control group, and three different concentrations of zinc sulfate-treated groups (100, 50, and 12.5 μmol·L^−1^). The protocol of zinc sulfate treatment was detailed in “2.2 section.” After 12 h, 24 h, 36 h, and 48 h of incubation, Marc−145 cells were harvested to detect PRRSV N gene copies. And after 48 h of incubation, Marc−145 cells were harvested to detect the relative expression of inflammation-related genes. After collecting cell samples from all groups, total RNA was extracted using TRIzol reagent (Catalog No. 9109, Takara, Beijing, China) following the manufacturer’s instructions. Total RNA concentrations were quantified using a NanoDrop 1,000 spectrophotometer. And cDNA was synthesized with the All-In-One 5 × RT Master Mix reverse transcription kit (Cat. No. G492, ABM, Shanghai, China). PRRSV N gene copies and the relative expression levels of inflammatory genes (*IL-6, IL-8, IL-10,* and *TNF-α*) were analyzed using an Applied Biosystems® 7,500 Real-Time PCR System (ABI 7500, Thermo Fisher Scientific, USA) with 2 × SYBR Green Master Mix (Cat. No. B21402, Bimake, Shanghai, China). All primer sequences were listed in Table 1. PRRSV N gene copies was quantitative analysis using standardized plasmid dilution series. The detailed protocols as follows: for the standard curve preparation: serial 10-fold dilutions (8 logarithmic gradients) of laboratory-preserved recombinant plasmid containing PRRSV N gene, PRRSV N gene-specific forward/reverse primers incorporated in the reaction system for qPCR amplification, and PRRSV load determination through comparative threshold cycle (Ct) analysis against the calibrated standard curve for absolute quantification. GAPDH served as the internal reference gene, and target gene expression levels were calculated using the 2^−ΔΔCt^ method. The experiment was repeated three times individually.

**Table 1:** Primer sequences for the qPCR.

Gene	Primer Sequences (5′-3′)
*GAPDH*	F: GTCAGTGGTGGACCTGACCT
	R: TGCTGTAGCCAAATTCGTTG
*IL-6*	F: CCAGCCACTGACCTCTTCAG
	R: TGTTTTCTGCCAGTGCCTCT
*IL-8*	F: GGTGCAGTTTTGCCAAGGAG
	R: TCCACAACCCTAGACACCCA
*IL-10*	F: GACCTCCGAGATGCCTTCAG
	R: TGATGTCTGGGTCGTGGTTC
*TNF-α*	F: GCTGCACTTTGGAGTGATCG
	R: CAGGCTTGTCACTTGGGGTT
*PRRSV N*	F: AGAAGCCCCATTTCCCTCTA
	R: CGGATCAGACGCACAGTATG

### 2.6 Western blot analysis of protein expression

Cells treatment and experimental grouping were explained in “2.2 section.” After 48 h of culture, the cells were collected and washed twice with cold PBS. Total cell extracts were prepared using RIPA lysis buffer (RIPA efficient lysis solution: protein phosphatase inhibitor: PMSF = 100: 1: 1, Code No. R0010, Solarbio, Beijing, China). And the mixture of cells and lysis buffer was vortexed for 15 s. Later, the samples were subjected to ultrasonication under ice-cold conditions with the following optimized parameters: power output: 200–300 W, pulse cycle: 2 s active sonication followed by 3 s cooling intervals, manual vortexing for 15 s at 10-min intervals, total duration: 30 min of cumulative processing. Later, the cell lysates were centrifuged at 12,000 × g for 15 min at 4°C, and the supernatants were collected for further experiments. Total protein concentration in cell extracts was measured using a BCA protein assay kit (Catalog No. P0012S, Beyotime Biotechnology, Shanghai, China).

200 μL of total protein supernatant was added to 50 μL of 5x protein loading buffer, and the samples were denatured by heating at 95°C for 10 min. Based on the total protein concentration measurement results, 30 μg of total protein sample was loaded per well (determined by prior quantification) for sodium dodecyl sulfate–polyacrylamide gel electrophoresis (SDS-PAGE) analysis. To standardize loading volumes, used the largest sample volume as a reference, and adjusted remaining samples to equivalent volumes using 1 × protein loading buffer. Prepare 1 × protein loading buffer by diluting 5 × buffer with cell lysis buffer (1:4 ratio). Denatured this mixture at 95°C for 10 min before use.

Subsequently, the cellular proteins were separated by 12% SDS-PAGE. Post-electrophoresis, excised target bands in alignment with the protein marker and transferred them on a polyvinylidene fluoride (PVDF) membrane using a Mini Trans-Blot Electrophoretic Transfer Cel and Module (Trans−Blot, Bio − Rad, America). The membrane was then blocked with 5% skim milk for 2 h at room temperature by following overnight incubation at 4°C with primary antibodies: anti-IL-6, anti-IL-8, anti-IL-10, anti-TNF-*α*, anti-p-IκBα, anti-p-p65, anti-Caspase3, anti-cleaved-Caspase3, anti-Bcl-2, and anti-Bax. Refer to Table 2 for antibody specifications (source, catalog numbers, dilution ratios).

**Table 2:** Antibody specifications (source, catalog numbers, dilution ratios).

Number	Antibody name	Manufacturer	Catalog number	Dilution ratio
1	TNF Alpha Monoclonal antibody	Proteintech	26,405-1-AP	1:1000
2	IL-6 Polyclonal antibody	Proteintech	21,865-1-AP	1:1000
3	IL-10 Monoclonal antibody	Proteintech	60,269-1-Ig	1:2000
4	CXCL8/IL-8 Polyclonal antibody	Proteintech	17,038-1-AP	1:500
5	IkB Alpha Polyclonal antibody	Proteintech	10,268-1-AP	1:5000
6	Phospho-IkB Alpha Monoclonal antibody	Proteintech	68,999-1-Ig	1:5000
7	NF-κB p65 Polyclonal antibody	Proteintech	10,745-1-AP	1:2000
8	Phospho-NF-kB p65 (Ser536) antibody	Affinity	AF2006	1:1000
9	GAPDH Monoclonal antibody	Proteintech	60,004-1-Ig	1:10000
10	Beta actin monoclonal antibody	Proteintech	66,009-1-Ig	1:10000
11	HRP-conjugated Goat Anti-Mouse IgG (H + L)	Proteintech	SA00001-1	1:10000
12	HRP-conjugated goat anti-rabbit IgG (H + L)	Proteintech	SA00001-2	1:10000

After washing three times with TBST, membranes were incubated for 1 h at 37°C with HRP-conjugated secondary antibodies Goat Anti-Mouse/Rabbit IgG (H + L). The sources, catalog numbers and dilution ratios are listed in Table 2. Target proteins were then detected using Meilunbio® FG Super-Sensitive ECL Luminescence Reagent (Code No. AR1171, Boster Biological Technology, USA), and the band images were captured with a chemiluminescence imaging system (ChemiDoc XRS+, Bio-Rad, USA).

Western blot band analysis was performed by using Image J software: the acquired images were imported into Image J and converted to 8-bit format. Background subtraction was performed using “Process” → “Subtract Background” with a rolling ball radius of 50 pixels (Light background option selected). Target protein bands were selected using the rectangular tool, and grayscale curves were generated via Analyze. A baseline was established using the straight-line tool, followed by automatic background subtraction. Finally, band intensity values were quantified to calculate relative protein expression levels.

### 2.7 Detection of ROS, MDA, CAT, and SOD levels using reagent kits

Cells treatment and experimental grouping were shown in “2.2 section.” ROS levels were measured using the Reactive Oxygen Species Assay Kit (Code No. S0033S, Beyotime Biotechnology, China), following the manufacturer’s instructions. The collected cells were incubated with 10 μmol·L^−1^ DCFH-DA probes for 30 min at 37°C in the dark. After washing three times with PBS, cellular fluorescence intensity was detected using a SpectraMax M5 microplate reader (excitation: 488 nm, emission: 525 nm). Relative ROS levels were expressed as fluorescence intensity.

MDA levels in all groups were measured using the Lipid Peroxidation MDA Assay Kit (Cat. No. S0131S, Beyotime, China). After washing the cells twice with PBS, cell lysis buffer was added. Then, the cells were centrifuged at 12,000 × g for 10 min, and the supernatants were collected for further use. The total protein concentration of cell lysates was measured using a BCA protein assay kit. MDA levels were measured according to the manufacturer’s protocol, and experimental details are summarized in Table 3. After preparing the reaction mixture as specified in Table 3, the solution was heated to 100°C for 15 min and then cooled to room temperature. After centrifugation at 1,000 × g for 10 min, 200 μL of the supernatant was transferred to a 96-well plate, and absorbance was measured at 532 nm using a SpectraMax M5 microplate reader (Molecular Devices, USA). In addition, a 1 mmol L^−1^ MDA standard solution was serially diluted to working concentrations of 1, 2, 5, 10, 20, and 50 μmol L^−1^ to generate the standard curve. MDA concentrations in samples were calculated from the standard curve and expressed as μmol mg^−1^ protein.

**Table 3:** MDA reaction system (mL).

List	Blank	Standard	Experimental sample
Lysis solution or PBS	0.1	/	/
Standard	/	0.1	/
Experimental sample	/	/	0.1
MDA detection working solution	0.2	0.2	0.2

SOD activity was measured using the Total Superoxide Dismutase Assay Kit (Code No. S0101S, Beyotime, China). After washing twice with PBS, the collected cells were resuspended in 200 μL PBS and subjected to three freeze–thaw cycles in liquid nitrogen and room temperature. Total cellular protein concentration was determined using a BCA protein assay kit. And SOD activity was measured based on the manufacturer’s protocol, with reaction system details provided in Table 4. After mixing, the reaction mixtures were incubated at 37°C for 20 min, and absorbance was measured at 450 nm using a SpectraMax M5 microplate reader (Molecular Devices, America). One unit of SOD activity was defined as the amount of enzyme required to inhibit its catalytic ability by 50%. The SOD activity calculation formula is provided in Equation 1:


(1) $$\begin{array}{l} S\mathrm{OD}\;\text{inhibition rater}\;(\%)=\\ \frac{(\text{AControl}-\text{ABlank})-\text{ASample}}{\text{AControl}-\text{ABlank}}\times 100\% \end{array}$$

$$\begin{array}{l} \mathrm{SOD}\;\text{activity}\;(U\cdot {\mathrm{mg}}^{-1})=\mathrm{SOD}\;\text{inhibition rater}\\ ÷50\%\times (0.24\;\mathrm{mL})÷(0.02\;\mathrm{mL})\\ ÷\text{Sample protein concentration} \end{array}$$

**Table 4:** SOD reaction system (mL).

List	Blank	Standard	Experimental sample
Lysis solution or PBS	0.1	/	/
Standard	/	0.1	/
Experimental sample	/	/	0.1
MDA detection working solution	0.2	0.2	0.2

The Catalase (CAT) activity in cells from all groups was measured using the CAT Activity Assay Kit (Code No. BC0205, Solarbio, China). After washing twice with PBS, the collected cells were resuspended in 300 μL of PBS and sonicated for 15 min in ice water. The parameters of ultrasonication were power output: 200–300 W, pulse cycle: 2 s active sonication followed by 3 s cooling intervals, manual vortexing for 15 s at 10-min intervals, total duration: 15 min of cumulative processing. The cell lysates were centrifuged at 5,000 × g for 10 min, and the supernatants were collected for further analysis. The total protein concentration in the supernatant was measured using a BCA protein assay kit. CAT activity was measured according to the manufacturer’s protocol. Adding 10 μL of supernatant and 190 μL of enzyme working solution in 96 well UV well, the mixture was immediately vortexed, and the initial absorbance (A_1_) was measured at 240 nm using a SpectraMax M5 microplate reader (Molecular Devices, USA). After 1 min, the second absorbance (A_2_) was measured at 240 nm. One unit of CAT activity is defined as the amount of enzyme required to decompose H_2_O_2_ in the substrate, causing the absorbance to decrease by 0.50–0.55 per second per gram of protein. The CAT activity was calculated as shown in Equation 2.


(2) $$\begin{array}{l} \mathrm{CAT}\;\text{activity}\;(U\cdot {\mathrm{mg}}^{-1}\;\text{protein})=\\ \left[\mathrm{\Delta A}\times \text{Total reaction volume}÷(\varepsilon \times d)\times 10^{6}\right]\\ ÷(\text{protein concentration}\times \text{sample volume})\\ ÷T=764.5\times \mathrm{\Delta A}÷\text{protein concentration} \end{array}$$

### 2.8 Detection of cell apoptosis by flow cytometry

Cell apoptosis levels were measured using an Annexin V-FITC/PI Apoptosis Detection Kit (Code No: MA0220, MeilunBio, China). Cell treatment and experimental groups were described in “Section 2.2.” After washing twice with cold PBS, cells were resuspended in 100 μL 1 × Binding Buffer working solution at a density of 1 × 10^6^ cells mL^−1^. Later, the cell suspension was mixed with 5 μL Annexin V-FITC and 5 μL PI working solution, vortexed briefly, and incubated in the dark for 15 min. After incubation, 400 μL of 1 × Binding Buffer was added and mixed. Apoptosis levels were then analyzed using the CytoFocus 421 flow cytometer (Beijing Zhizhen Biotechnology Co., Ltd., China).

Flow cytometric analysis was performed under the following optimized parameters: Fluorescence channels: FITC (490 nm/525 nm), PI (535 nm/615 nm). Cell gating strategy: Select the live cell population (P1 gate) in the FSC-A/SSC-A scatter plot, set the FITC/PI fluorescence baseline using unstained cells, adjusted the fluorescence compensation using the Annexin V single staining group and PI single staining group, and performed a four-quadrant analysis of the P1 gate cells on Annexin V-FITC (X axis) vs. PI (Y axis). Early apoptosis: Annexin V^+^/PI^−^ (lower right quadrant), Late apoptosis: Annexin V^+^/PI^+^ (upper right quadrant). Cells treated with a 95°C heat shock for 10 s serve as positive controls, with each sample acquiring ≥10,000 cells within the P1 gate. Data are analyzed using FlowJo 10.8 software, and experiments are independently repeated three times (*n* = 3).

### 2.9 Immunofluorescence detection of PRRSV N protein expression in Marc-145 cells

The experiment groups included a blank control group, a PRRSV-infected control group, and three different concentrations of zinc sulfate-treated groups (100, 50, and 12.5 μmol·L^−1^). The protocol of zinc sulfate treatment was detailed in “2.2 section.” And after 48 h of incubation, Marc−145 cells were harvested to detect the expression of PRRSV N protein by indirect immunofluorescence assay using immunofluorescence microscopy (IX81-F72FL/PH, Olympus, Japan). The cells were washed 3 × with PBS (3 min/wash) and fixed with 4% paraformaldehyde for 15 min, then wash for 3 × with PBS. Subsequently, the cells were permeabilized with 0.1% Triton X-100 for 15 min, followed by PBS washes for 3×. Later, the samples were blocked with 5% BSA at 37°C for 1 h, and incubated with anti-PRRSV nucleocapsid primary antibody (1:500 dilution in PBS, Genetex, USA) at 4°C in dark for overnight, then washed 3 × with PBS. Next, the cells were stained with CoraLite®488-conjugated goat anti-rabbit IgG (H + L) (1:500 dilution in PBS, Cat No. SA00013-2, Proteintect, Wuhan Sanying, China) at 37°C in dark for 1 h, then washed for 3 times with PBS. Finally, counterstain nuclei with DAPI in dark for 5 min and image using immunofluorescence microscopy (IX81-F72FL/PH, Olympus, Japan). The infection rates was quantified by the percentage of fluorescent cells using Image J: (PRRSV+ cells/DAPI+ cells) × 100%.

### 2.10 Statistical analysis

All statistical analyses were performed using GraphPad Prism software 8.0 (GraphPad Software, Inc. California, USA). Statistical significance was determined using one-way ANOVA followed by Dunnett’s test for comparisons with the PRRSV-infected control group. Furthermore, all statistical analyses have undergone rigorous re-evaluation, confirming: Brown-Forsythe test: *p* > 0.1 and Shapiro–Wilk test: *p* > 0.05. A *p*-value <0.05 was considered to be statistical significance and indicated that the difference was significant. And a *p*-value <0.01, 0.001, or 0.0001 indicated that the difference were extremely significant. The experiments are independently repeated three times (*n* = 3) and the data were expressed as mean±standard errors.

## 3 Results

### 3.1 Zinc sulfate inhibits the PRRSV replication in Marc−145 cells

**Figure 1:**
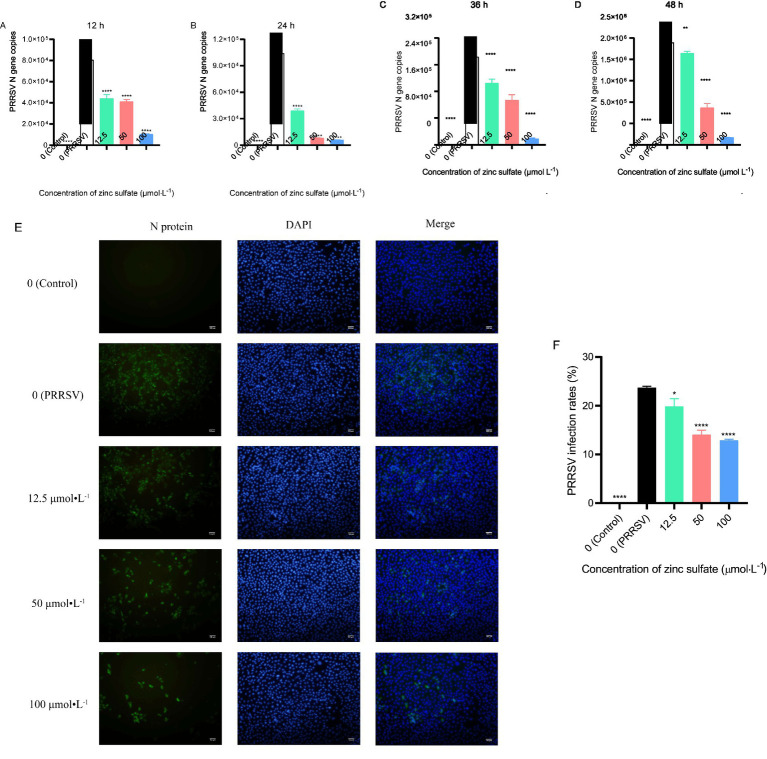
Zinc sulfate inhibits PRRSV replication at 12 h, 24 h, 36 h, and 48 h. The relative expression level of PRRSV N gene in PRRSV-infected cells were detected followed by treatment with or without zinc sulfate at 12 h, 24 h, 36 h, and 48 h by qPCR **(A–D)**. The expression of PRRSV N protein was detected at 48 h by indirect immunofluorescence assay in cells PRRSV-infected and exposed to a concentration gradient of zinc sulfate **(E)**, PRRSV particles was labeled with green fluorescence and the nucleus was labelled with blue fluorescence, and scale bar = 100 μm (100× magnification). The rate of PRRSV-infected cells was quantified by the percentage of fluorescent cells using Image J software **(F)**. Statistical significance was determined using one-way ANOVA followed by Dunnett’s test for comparisons with the PRRSV-infected control group. Data represent mean ± SEM of n=3 biological replicates. Significant differences compared with the PRRSV-infected control group are denoted by * (*p* < 0.05), ** (*p* < 0.01), *** (*p* < 0.001), and **** (*p* < 0.0001). No significant differences compared with the PRRSV-infected control group are denoted by ns (*p* > 0.05).

To investigate the role of zinc sulfate in PRRSV infection, the expression level of the PRRSV N gene was detected by qPCR, and the expression of PRRSV N protein was analyzed by indirect immunofluorescence assay (Figures 1A–F). The results demonstrated that, compared to the PRRSV-infected group, PRRSV N gene copies exhibits a significant reduction (*p* < 0.05) across zinc sulfate treatment groups at 12, 24, 36, and 48 h post-infection (Figures 1A–D). Immunofluorescence analysis revealed that, compared to the PRRSV-infected group, zinc sulfate treatments significantly reduced the number of PRRSV-infected cells at 48 h post-infection (*p* < 0.05, *p* < 0.00001) (Figures 1E,F). Moreover, within the concentration range of 12.5–100 μmol L^−1^, a clear dose-dependent inhibitory effect was observed.

### 3.2 Screening of zinc sulfate-mediated anti-PRRSV target genes

**Figure 2:**
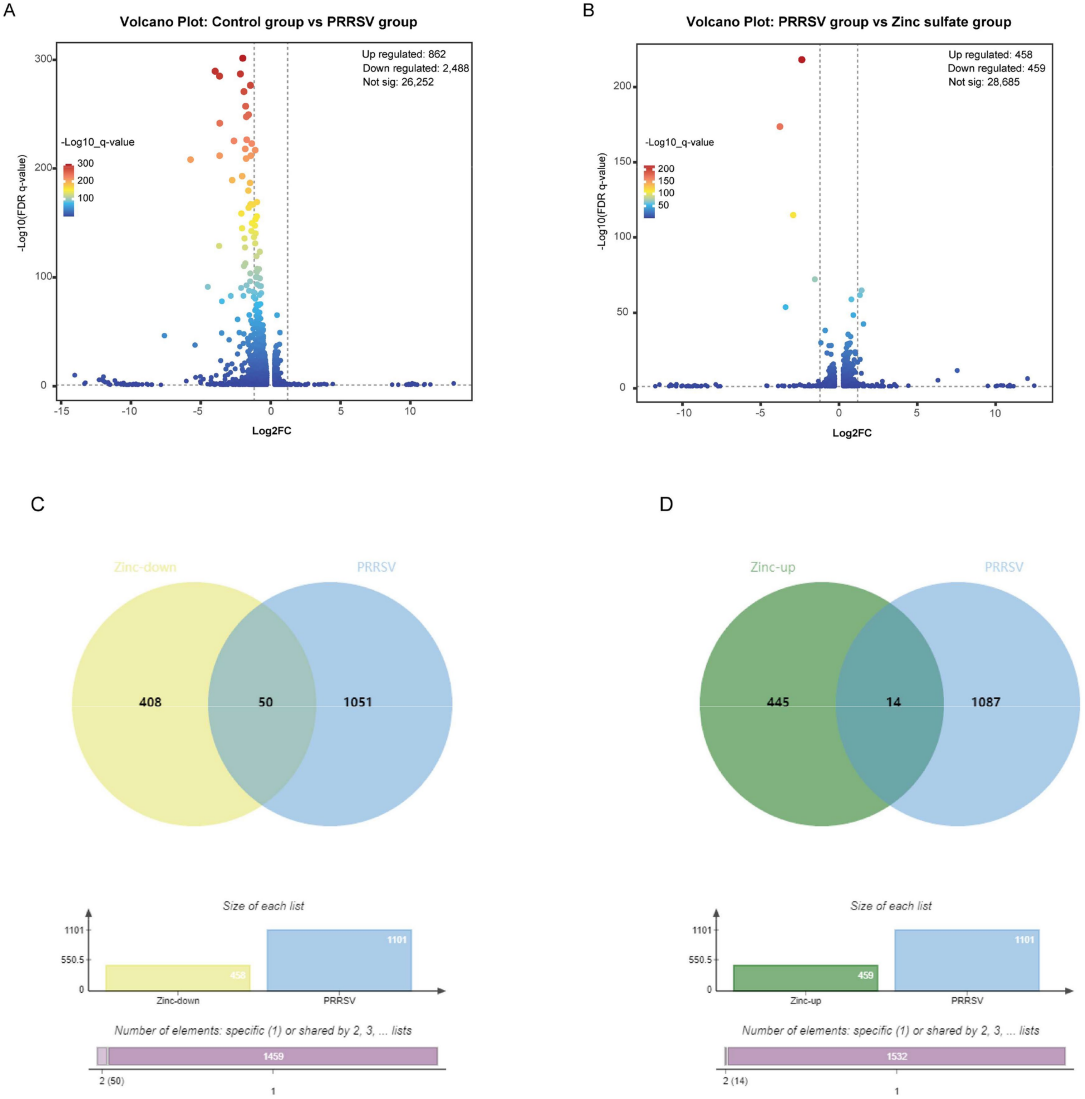
Differentially expressed genes in Marc-145 cells with PRRSV inoculation and/or zinc sulfate treatment. The volcano plots of differentially expressed genes profiling in each group **(A,B)**, and the Veen analysis plots of up-regulated and down-regulated genes in each group **(C,D)**. The bar charts in **(C,D)** provide complementary visualization, with the “Specific (1)“bar representing 1,459 specific genes in zinc treatment downregulated genes and PRRSV-associated disease-related genes, and the “Shared by 2″ column indicates the 50 genes exhibiting differential expression patterns in both zinc treatment downregulated genes and PRRSV-associated disease-related genes in **(C)**, and with the “Specific (1)” bar representing 1,532 specific genes in zinc treatment upregulated genes and PRRSV-associated disease-related genes, and the “Shared by 2″ column indicates the 14 genes exhibiting differential expression patterns in both zinc treatment upregulated genes and PRRSV-associated disease-related genes in **(D)**.

A high-throughput RNA transcriptomic sequencing analysis was performed to elucidate the molecular mechanism underlying Zinc sulfate-mediated anti-PRRSV activity. Marc-145 cells were treated with 100 μmol·L^−1^ zinc sulfate for 48 h post-PRRSV infection, followed by comprehensive gene expression profiling. Differential gene expression analysis among the control group, PRRSV infection group, and zinc sulfate treatment group were completed and displayed by the volcano plot visually (Figures 2A,B). Compared with the control group, a total of 3,350 significantly differentially expressed genes (DEGs; *p* < 0.05, |log₂FC| > 1.2) are identified in the PRRSV infection group (Figure 2A). Among these 2,488 genes were downregulated and 862 genes were upregulated. Compared with the PRRSV infection group, 917 DEGs (*p* < 0.05, |log₂FC| > 1.2) exhibited significant alterations upon zinc sulfate treatment (Figure 2B). Simultaneously, there were 459 downregulated genes and 458 upregulated genes among 917 differential gene expression.

To identify overlapping targets between the zinc sulfate-treated and PRRSV-infected groups, Venn analysis was performed to compare the 459 downregulated and 458 upregulated genes from the zinc sulfate-treated group against 1,101 PRRSV-associated disease-related genes. Visualization was implemented using the Bioinformatics Venn platform (Figures 2C,D) and 50 co-downregulated and 14 co-upregulated genes were exhibited in Table 5. The results demonstrated that there were 14 co-upregulated genes (Figure 2C) and 50 co-downregulated genes (Figure 2D), the former potentially associated with host defense mechanisms, and the later likely involved in viral suppression of host cellular processes.

**Table 5:** 50 co-downregulated and 14 co-upregulated genes.

50 downregulated genes			14 upregulated genes
DDIT3	MMP9	SOX9	CDKN3
CNR1	JUN	ABCC9	ADORA1
NFKBIA	FOXO1	IRF1	CDC25C
PI3	ICAM1	MAF	ADAMTS13
NTF3	FCGR3A	CD79A	PRKAA2
EGR1	NR4A1	IL6	CDKN1C
NFKB1	HBEGF	C3	BIRC5
FOS	BMP2	LTF	GABRA1
SOD2	ATP8B1	CSF1	TTN
SERPINA1	ATF3	CSF2	UCN
SERPINA3	CXCL1	MYC	COL7A1
RARA	CXCL8	ADORA2A	ANGPT1
TNF	PECAM1	BHLHE40	CLDN2
PLAU	TNFSF10	STAT3	DHFR
PLAT	CCL2		
PRF1	NOD2		
AGRN	ADRB1		
GLRX	ADM		

### 3.3 PPI network construction for the 50 co-downregulated genes and 14 co-upregulated genes

**Figure 3:**
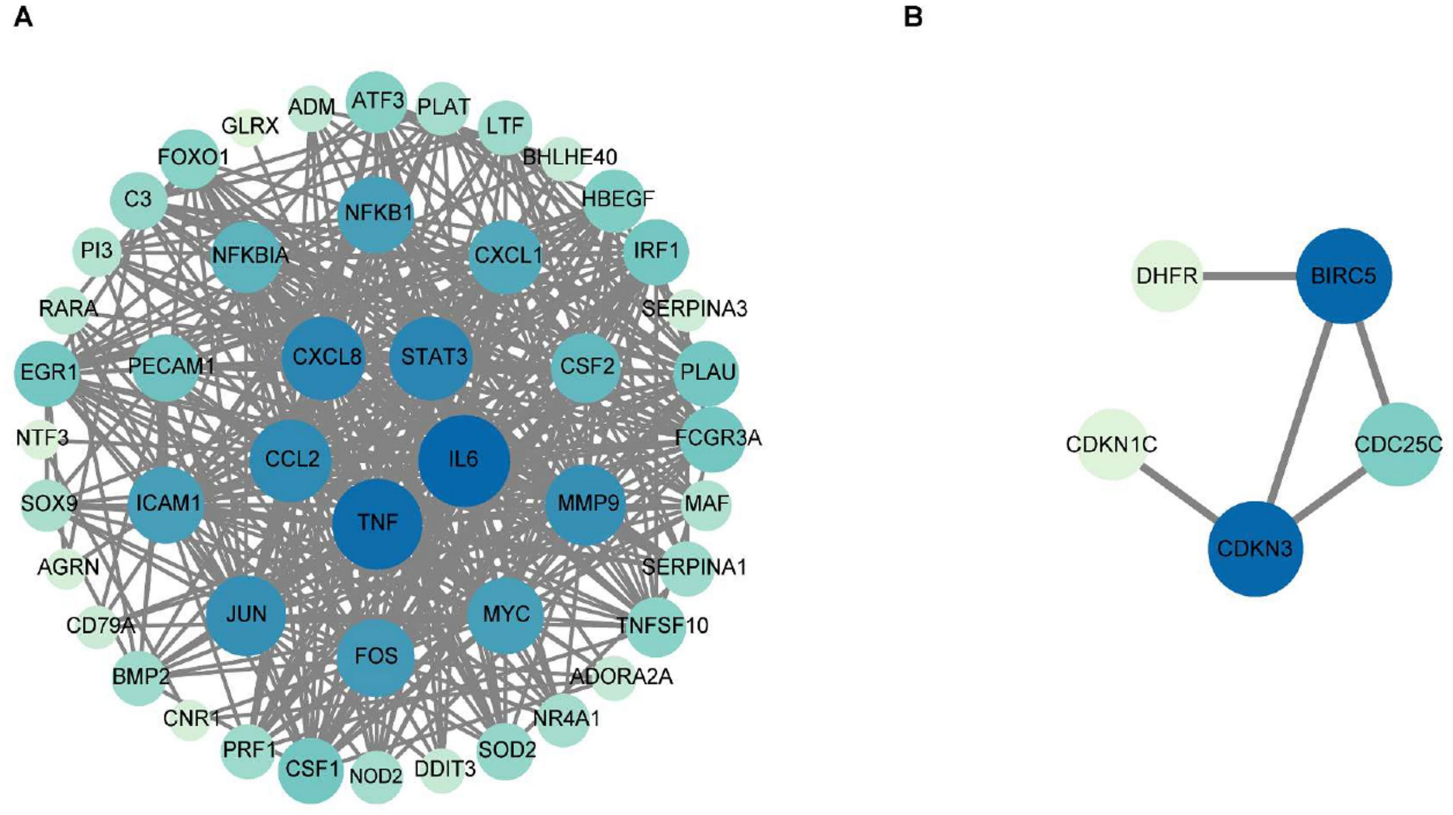
The protein–protein interaction network for the 50 co-downregulated and 14 co-upregulated genes. Protein interaction network diagram of 50 co-downregulated intersection targets and the 15 core target proteins **(A)**, and protein interaction network diagram of 14 co-upregulated intersection targets and the 5 core target proteins **(B)**. The chromatic gradation of nodes within the PPI network demonstrates a direct proportionality to their respective degree values. As illustrated in **(A)**, nodes exhibiting deeper pigmentation denote the 15 hub genes bearing degree values exceeding threshold 22, strategically positioned within the innermost and intermediate concentric circles, whereas their lightly tinted counterparts with degree values below threshold 22 occupy the peripheral circle. In **(B)**, the PPI network visualization identifies two key hub genes (dark blue nodes) with degree values of 3, along with three additional hub genes (light-colored nodes) exhibiting degree values below 3.

To delineate functional relationships among the 50 co-downregulated genes, a PPI network was constructed (Figure 3A). Network analysis identifies 15 high-degree hub genes, functionally stratified into four key categories: pro-inflammatory mediators (TNF, IL6, CXCL8, CCL2, CXCL1, CSF2), signal transduction regulators (STAT3, JUN, MYC, NFKBIA), extracellular matrix modulators (MMP9, PLAU), and cell adhesion molecules (ICAM1, PECAM1) (Table 5).

To investigate functional relationships among the 14 co-upregulated genes, a PPI network was established (Figure 3B). Key hub genes identified through network analysis include cell-cycle regulators (BIRC5, CDKN3, CDKN1C, and CDC25C) and metabolic enzyme (DHFR).

### 3.4 GO functional and KEGG enrichment analysis of 50 downregulated and 14 upregulated intersection targets

**Figure 4:**
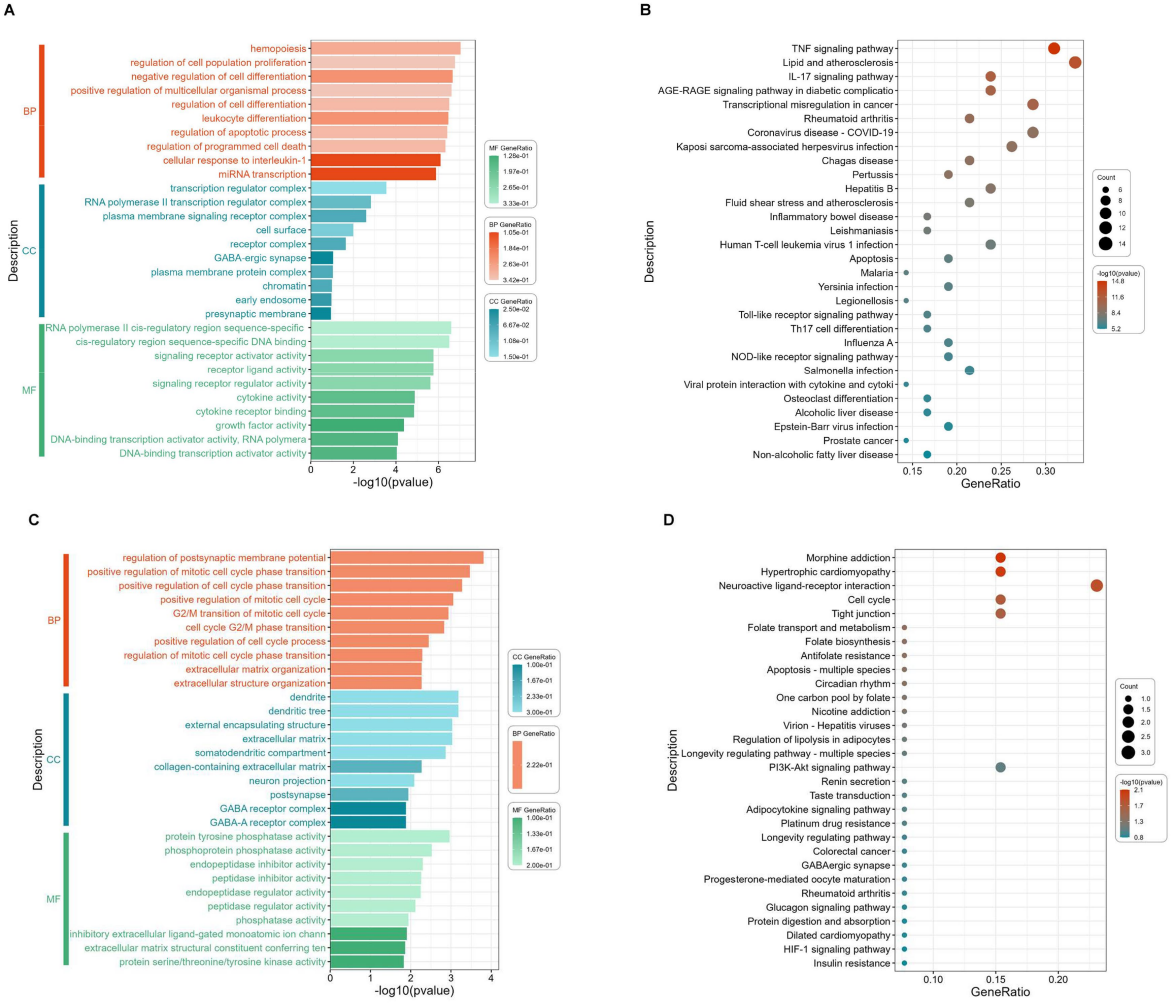
GO terms enrichment and KEGG pathway enrichment analysis of the 50 downregulated and 14 upregulated intersection targets. The most significant enriched GO terms (the top 10 from each ontology BP, CC, and MF, *p* < 0.05) among the 50 downregulated intersection targets **(A)**, the most significant enriched KEGG pathways (top 30, *p* < 0.05) among the 50 downregulated intersection targets **(B)**, the most significant enriched GO terms (the top 10 from each ontology BP, CC, and MF, *p* < 0.05) among the 14 upregulated intersection targets **(C)**, and the most significant enriched KEGG pathways (top 30, *p* < 0.05) among the 14 upregulated intersection targets **(D)**.

To elucidate the biological functions and associated signaling pathways of the 50 downregulated intersection targets, comprehensive GO and KEGG pathway enrichment analyses were conducted (Figures 4A,B). As show in Figure 4A, GO functional analysis of 50 downregulated intersection targets exhibits that the top BP include cell fate decision, programmed cell death, immune and inflammatory response, and the major CC of 50 downregulated DEGs can be classified into the membrane, the receptor complex on the membrane, and the regulator complex related to transcription, as well, the MF was mainly related with “factor activity,” such as cytokine, receptor ligand, and growth factor. As shown in Figure 4B, 50 downregulated intersection targets were involved in 90 signaling pathways, including the TNF signaling pathway, Lipid and atherosclerosis, IL-17 signaling pathway, and AGE-RAGE signaling pathway in diabetic complications, which were well enriched.

To elucidate the biological functions and associated signaling pathways of the 14 upregulated intersection targets, comprehensive GO and KEGG pathway enrichment analyses were conducted (Figures 4C,D). As shown in Figure 4C, GO functional analysis of 14 upregulated intersection targets shows that the BP mainly enriched in cell cycle, extracellular structure organization, and membrane potential, and the CC can be classified into extracellular matrix, GABA-receptor complex, and dendrite, similar to 50 downregulated DEGs, the MF also focuses on “activity,” including phosphatase activity, inhibitor activity, and regulator activity. As demonstrated in Figure 4D, KEGG analysis of 14 upregulated intersection targets exhibited that morphine addiction, hypertrophic cardiomyopathy, neuroactive ligand-receptor interaction, cell cycle, and tight junction were well-enriched.

### 3.5 Interaction network construction of zinc sulfate-core target proteins-pathways

**Figure 5:**
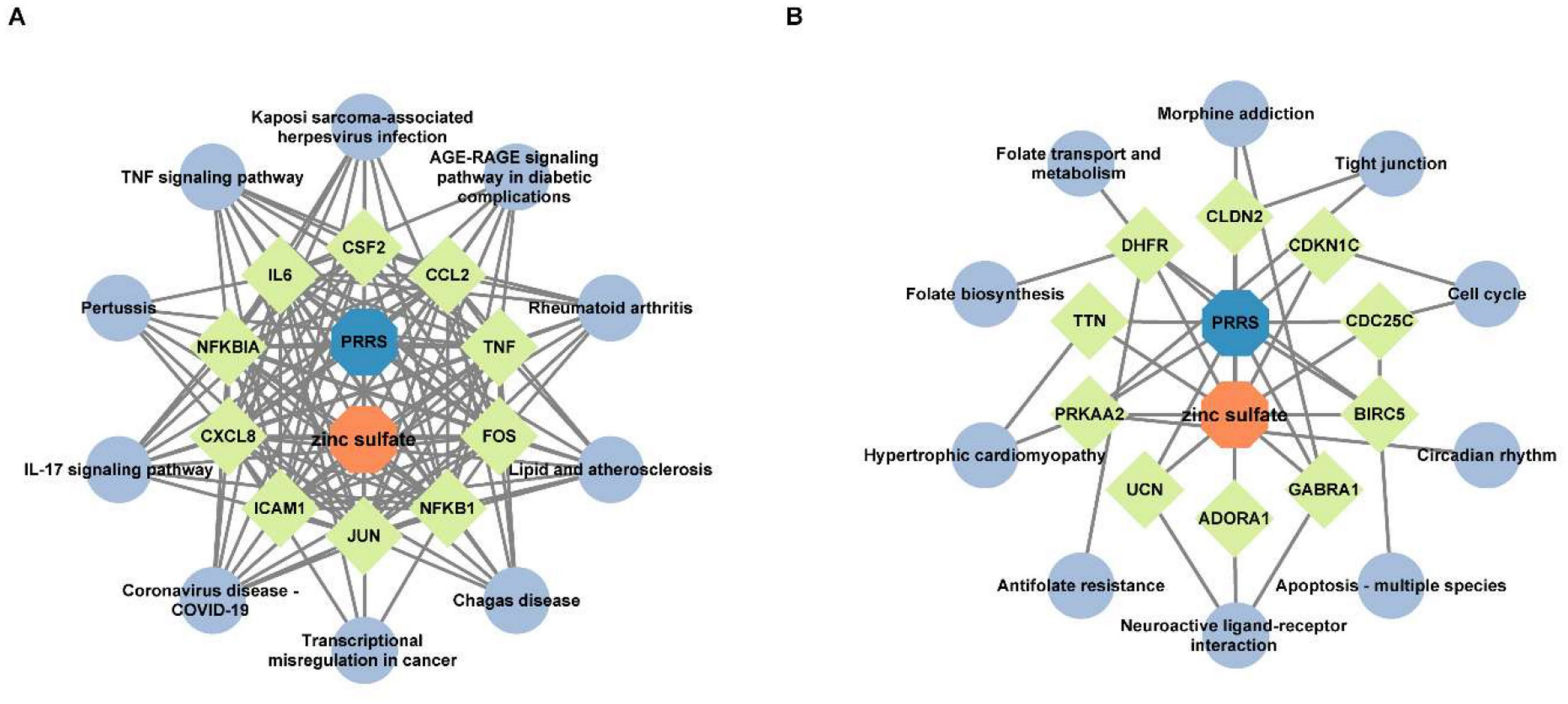
The interaction network of zinc sulfate-core target proteins-pathways. The interaction network of the top 10 hub genes with the top 10 KEGG pathway among 50 downregulated intersection targets **(A)**, and the interaction network of the top 10 hub genes with the top 10 KEGG pathway among 14 upregulated intersection targets **(B)**. The blue, orange, chartreuse, and grayish blue colors represent PRRS disease, zinc sulfate, the core targets (the top 10 hub genes in betweenness centrality and degree), and the signal pathways (the top 10 KEGG pathways), respectively.

To elucidate the molecular mechanism by which zinc sulfate modulates key downregulated targets against PRRSV infection, an integrated regulatory network was constructed using Cytoscape 3.10.0 (Figure 5A). The resulting zinc sulfate-PRRSV-core target-signaling pathway interaction model are comprised of 22 nodes (including 10 key targets and 10 enriched pathways) and 142 edges (representing functional interactions). Network analysis identified 10 cross-pathway hub genes, which involve in cytokine mediators (IL6, CXCL8, TNF, CCL2, CSF2), transcriptional regulators (JUN, FOS, NFKB1, NFKBIA), and adhesion molecules (ICAM1). The most significantly enriched pathways included the TNF signaling pathway, Lipid and atherosclerosis, Kaposi sarcoma-associated herpesvirus infection, AGE-RAGE signaling pathway in diabetic complications, Rheumatoid arthritis, Chagas disease, Transcriptional misregulation in cancer, Coronavirus disease-COVID-19, IL-17 signaling pathway, and Pertussis. These results indicate that zinc sulfate can exerts its anti-PRRSV effects by coordinately downregulating core genes such as IL6 and TNF and participates in the inflammatory regulation process related to PRRSV infection through a multi-target/ multi-pathway framework.

To elucidate the regulatory mechanism of zinc sulfate on upregulated core targets in PRRSV infection, we integrated 10 significantly enriched pathways and 10 target genes. Using Cytoscape 3.10.0, we constructed the zinc sulfate-PRRSV-upregulated core target-pathway interaction network (Figure 5B). The network is comprised of 22 nodes (including 10 key targets and 10 enriched pathways) and 38 edges (representing functional interactions). The results showed that 10 cross-pathway core upregulated hub genes mainly involved in cell cycle/apoptosis regulators: BIRC5, CDC25C, CDKN1C, metabolic/structural genes: DHFR, TTN, PRKAA2, neural/receptor proteins: GABRA1, ADORA1, UCN, tight junction component: CLDN2 and the top enriched pathways were circadian rhythms, apoptosis-multiple species, neuroactive ligand-receptor interaction, antifolate resistance, hypertrophic cardiomyopathy, folate biosynthesis, folate transport and metabolism, morphine addiction, tight junction, and cell cycle. These results demonstrate that zinc sulfate can exerts anti-PRRSV effects by coordinately regulating apoptosis-related core genes including BIRC5, CDC25C, CDKN1C and influencing apoptotic pathways associated with PRRSV infection.

### 3.6 Zinc sulfate suppresses PRRSV proliferation by abating oxidative stress reaction

**Figure 6:**
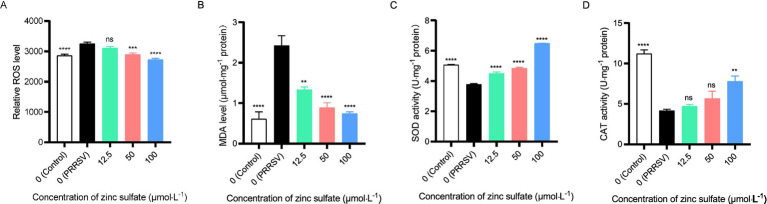
Zinc sulfate alleviates oxidative stress biomarker elicited by PRRSV. ROS level in PRRSV-infected cells was detected followed by treatment with or without zinc sulfate at 48 h using reactive oxygen species assay kit, the cells were incubated with 10 μmol·L^−1^ DCFH-DA probes for 30 min at 37°C in the dark, after washing three times with PBS, cellular fluorescence intensity was detected using a microplate reader. **(A)**, MDA level in PRRSV-infected cells was analyzed followed by treatment with or without zinc sulfate at 48 h using MDA assay kit, and MDA concentrations in samples were calculated from a standard curve **(B)**, SOD enzyme activity in PRRSV-infected cells was determined followed by treatment with or without zinc sulfate at 48 h using T-SOD assay kit, and the mixtures of supernatant of sample and enzyme working solution were incubated at 37°C for 20 min, and absorbance was measured at 450 nm using a microplate reader **(C)**, and CAT enzyme activity in PRRSV-infected cells was measured followed by treatment with or without zinc sulfate at 48 h using CAT assay kit, and the absorbance of mixture with the supernatant of sample and enzyme working solution was detect at 240 nm using a microplate reader **(D)**. Statistical significance was determined using one-way ANOVA followed by Dunnett’s test for comparisons with the PRRSV-infected control group. Data represent mean ± SEM of n = 3 biological replicates. Significant differences compared with the PRRSV-infected control group are denoted by *p < 0.05, **p < 0.01, ***p < 0.001, and ****p < 0.0001. No significant differences compared with the PRRSV-infected control group are denoted by ns (*p* > 0.05).

To elucidate the underlying mechanism of zinc sulfate against PRRSV, we analyzed oxidative stress biomarkers, including ROS levels, MDA content, and the enzymatic activities of SOD and CAT in cells PRRSV-infected and treated with varying concentrations of zinc sulfate (Figures 6A–D). The results revealed that compared to the non-treated cells group, the levels of ROS in PRRSV infected groups exhibits an extremely significant increase (*p* < 0.0001); while, compared to PRRSV infected groups, the ROS levels in 50 and 100 μmol·L^−1^ zinc sulfate treated groups display an extremely significant reduction (*p* < 0.001, *p* < 0.0001) (Figure 6A). For the detection of MDA, the MDA level in PRRSV infected group extremely significantly elevates compared with the non-treated cells group; meanwhile, compared with the PRRSV infected group, the MDA levels in the 50 and 100 μmol·L^−1^ zinc sulfate treated groups were significantly reduced (*p* < 0.001, *p* < 0.0001) (Figure 6B). It can be found that compared to the non-treated cells group, the SOD activity was significantly reduced for the PRRSV infected group (*p* < 0.0001); but compared with PRRSV infected group, the SOD activity of 12.5, 50, 100 μmol·L^−1^ zinc sulfate treated groups extremely significantly elevated (*p* < 0.0001) (Figure 6C). In addition, compared to the non-treated group, the CAT activity in PRRSV infected group was significantly reduced (*p* < 0.0001); and compared with PRRSV infected group, only 100 μmol·L^−1^ zinc sulfate treated groups exhibit the significantly elevating trend (*p* < 0.01) and 12.5, 50 μmol·L^−1^ zinc sulfate treated groups have no significant different (Figure 6D).

### 3.7 Zinc sulfate inhibits PRRSV proliferation by reducing inflammatory response

**Figure 7:**
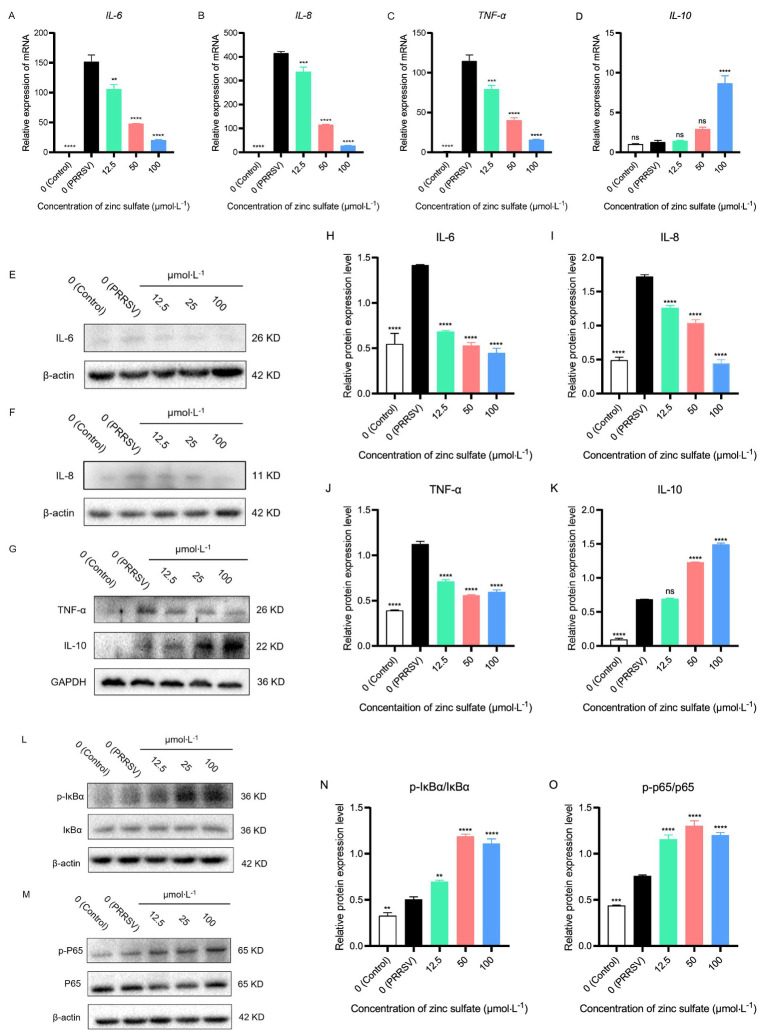
Zinc sulfate reduced the gene and protein expression of key pro-inflammatory (IL-6, IL-8, TNF-*α*) cytokines, while elevating anti-inflammatory (IL-10) cytokine and the protein expression related to NF-κB signal pathway in cells PRRSV-infected and exposed to a concentration gradient of zinc sulfate. The relative gene expression level of IL-6, IL-8, TNF-α, and IL-10 in PRRSV-infected cells were detected followed by treatment with or without zinc sulfate at 48 h by qPCR **(A–D)**. The protein expression of IL-6, IL-8, TNF-α, and IL-10 in PRRSV-infected cells were detected followed by treatment with or without zinc sulfate at 48 h by WB **(E–G)**. The band intensities of IL-6, IL-8, TNF-α, and IL-10 protein expression were quantified by analyzing gray values using Image J software **(H–K)**. The protein expression of p-IκBα, IκBα, p-p65, and p65 in PRRSV-infected cells were detected followed by treatment with or without zinc sulfate at 48 h by WB **(L,M)**. The band intensities of p-IκBα, IκBα, p-p65, and p65 protein expression were quantified by analyzing gray values using Image J software, and the relative protein expression level of p-IκBα/IκBα and p-p65/p65were analyzed **(N,O)**. Statistical significance was determined using one-way ANOVA followed by Dunnett’s test for comparisons with the PRRSV-infected control group. Data represent mean ± SEM of *n* = 3 biological replicates. Significant differences compared with the PRRSV-infected control group are denoted by *p < 0.05, **p < 0.01, ***p < 0.001, and ****p < 0.0001. No significant differences compared with the PRRSV-infected control group are denoted by ns (*p* > 0.05).

To evaluate the inflammatory-modulatory mechanism of zinc sulfate against PRRSV infection, the relative gene expression levels of key pro-inflammatory (IL-6, IL-8, TNF-*α*) and anti-inflammatory (IL-10) cytokines were quantified by qRT-PCR in cells PRRSV-infected and exposed to a concentration gradient of zinc sulfate (Figures 7A–D). The results showed that compared to the blank group, the relative expressions of *IL-6*, *IL-8*, and *TNF-α* gene display a highly significant increase (*p* < 0.0001) in PRRSV-infected group; and compared to PRRSV-infected group, the relative expressions of *IL-6*, *IL-8*, and *TNF-α* gene show a significantly downward trend (*p* < 0.01, *p* < 0.001, *p* < 0.0001) in 12.5, 50 and 100 μmol·L^−1^ zinc sulfate treated groups (Figures 7A–C) and appeared a dose-effect relationship between the relative expressions of gene and the concentration of zinc sulfate. For the *IL-10* gene, compared to the blank group, no significant change observed in PRRSV-infected group; and the relative gene expression of *IL-10* only in 100 μmol·L^−1^ zinc sulfate treated group exhibited extremely significant ascension (*p* < 0.0001) compared with PRRSV-infected group (Figure 7D).

Following a 48 h PRRSV infection in Marc-145 cells, protein expression of IL-6, IL-8, IL-10, and TNF-*α* was analyzed by western blotting in the cells group, PRRSV-infected group, and exposed to a concentration gradient of zinc sulfate group (Figures 7E–G). Correspondingly, the band intensities were quantified by analyzing gray values using Image J software (Figures 7H–K). The results demonstrated that compared with the control group, the protein expression levels of IL-6, IL-8, TNF-α, and IL-10 in PRRSV-infected group were significantly increased (*p* < 0.0001) (Figures 7H–K); compared with PRRSV-infected group, and the protein expression levels of IL-6, IL-8, and TNF-α were significantly reduced in the 12.5, 50, and 100 μmol·L^−1^ zinc sulfate treatment groups (*p* < 0.0001) (Figures 7H–J), while the protein expression level of IL-10 was significantly increased in the 50 and 100 μmol·L^−1^ zinc sulfate treatment groups (*p* < 0.0001) (Figure 7K). The results illustrate that PRRSV infection induces gene and protein expression of IL-6, IL-8, TNF-α, and IL-10 in cells, while zinc sulfate treatment effectively reversed the PRRSV-induced upregulation of IL-6, IL-8, and TNF-α, and furtherly potentiated IL-10 expression.

After 48 h of PRRSV infection in Marc-145 cells, zinc sulfate was used to detect the protein expression levels of IκBα, p-IκBα, p65, and p-p65 using western blot (Figures 7L,M). And the relative protein expression of p-IκBα/IκBα and p-p65/p65 were analyzed (Figures 7N,O). The results showed that compared with the blank group, the expression levels of p-IκBα and p-p65 proteins were significantly increased in the PRRSV group (*p* < 0.01, *p* < 0.001, *p* < 0.0001) (Figures 7L,M); Compared with the PRRSV group, the expression levels of p-IκBα and p-p65 proteins were significantly increased in the 12.5, 50, and 100 μmol·L^−1^ zinc sulfate treatment groups (*p* < 0.01, *p* < 0.0001) (Figures 7N,O). The results showed that PRRSV infection promotes the expression of p-IκBα and p-p65 in cells, while zinc sulfate treatment further enhances the expression levels of these two proteins.

### 3.8 Zinc sulfate hinders PRRSV proliferation by restraining cell apoptosis

**Figure 8:**
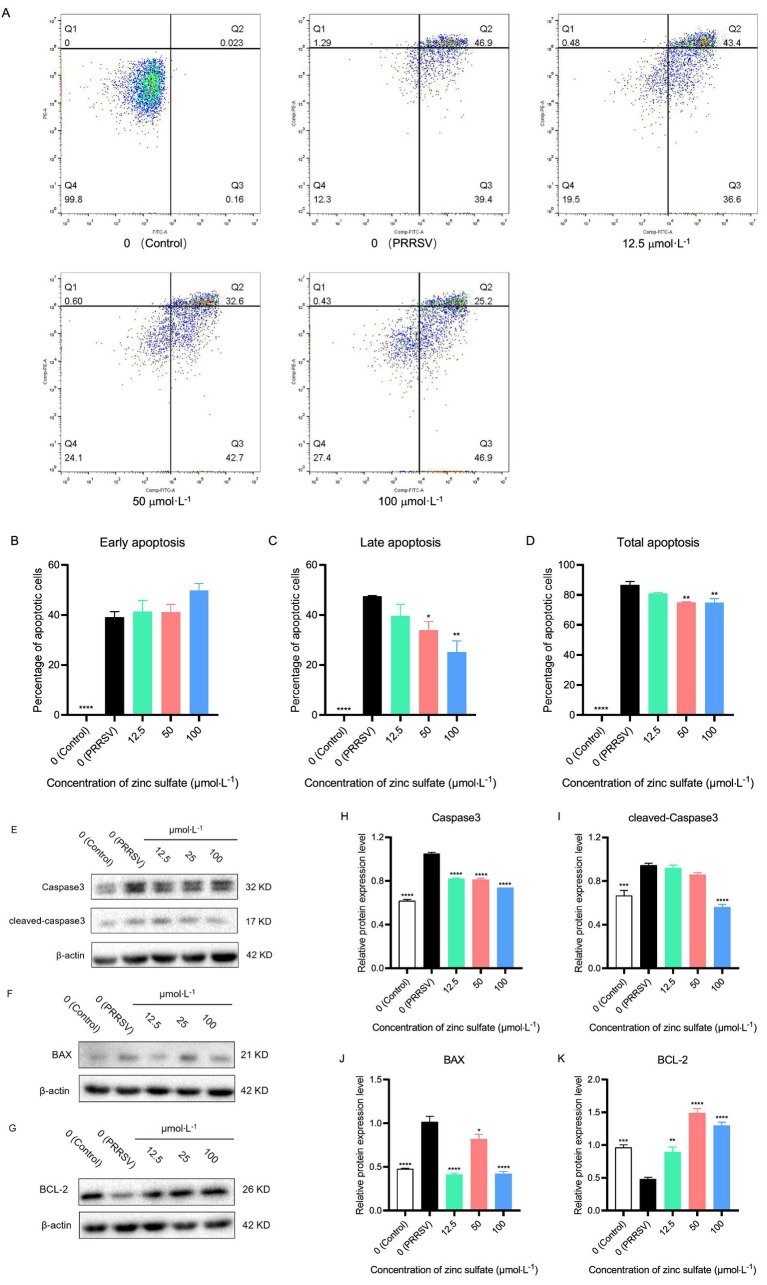
Zinc sulfate suppresses apoptosis in cells PRRSV-infected and exposed to a concentration gradient of zinc sulfate. The apoptosis in PRRSV-infected cells was detected followed by treatment with or without zinc sulfate at 48 h by flow cytometry, and perform a four-quadrant analysis, Q1 represents mechanical necrosis, Q2 represents late apoptosis, Q3 represents early apoptosis, and Q4 represents live cells **(A)**. Early apoptosis ratio, late apoptosis ratio, and total apoptosis ratio were analyzed **(B–D)**. The protein expression of Caspase 3, cleaved-Caspase 3, Bax, and Bcl-2 in PRRSV-infected cells were detected followed by treatment with or without zinc sulfate at 48 h by WB **(E–G)**. The band intensities of Caspase 3, cleaved-Caspase 3, Bax, and Bcl-2 protein expression were quantified by analyzing gray values using Image J software **(H–K)**. Statistical significance was determined using one-way ANOVA followed by Dunnett’s test for comparisons with the PRRSV-infected control group. Data represent mean ± SEM of *n* = 3 biological replicates. Significant differences compared with the PRRSV-infected control group are denoted by *p < 0.05, **p < 0.01, ***p < 0.001, and ****p < 0.0001. No significant differences compared with the PRRSV-infected control group are denoted by ns (*p* > 0.05).

After 72 h of treatment with zinc sulfate on PRRSV-infected Marc-145 cells, the cells were collected and stained with Annexin V-FITC/PI cell apoptosis assay kit, and detected by flow cytometry (Figure 8A). And the early, late, and total apoptosis rates were analyzed in each groups (Figures 8B–D). The results showed that compared with the control group, the early, late, and total cell apoptosis rates are significantly increased in the PRRSV group (*p* < 0.0001) (Figures 8B–D). Compared with the PRRSV group, the late-stage cell apoptosis rate and total cell apoptosis rate are significantly reduced in the 50 and 100 μmol·L^−1^ zinc sulfate groups (*p* < 0.05, *p* < 0.01, *p* < 0.0001) (Figures 8C,D). The results revealed that PRRSV infection induces apoptosis in Marc-145 cells, while zinc sulfate treatment inhibits PRRSV-induced apoptosis, predominantly in late-stage cells.

After 48 h of treatment with zinc sulfate on PRRSV-infected Marc-145 cells, the cells were collected and total protein was extracted. Western blot was used to detect the expression levels of caspase3, cleaved-caspase3, Bcl-2, and Bax proteins (Figures 8E–G). And their gray values were analyzed (Figures 8H–K). The results show that compared with the control group, the expression levels of caspase3, cleaved-caspase3, and Bax proteins in the PRRSV group were significantly increased (*p* < 0.001, *p* < 0.0001), while the expression level of Bcl-2 protein is significantly decreased (*p* < 0.001) (Figures 8H–K); Compared with the PRRSV group, the relative expression level of caspase3 was significantly reduced in the 12.5, 50, and 100 μmol·L^−1^ zinc sulfate treatment groups (*p* < 0.0001) (Figure 8H), and the expression level of cleaved-Caspase3 protein is significantly reduced in the 100 μmol·L^−1^ zinc sulfate treatment group (*p* < 0.001) (Figure 8I); Compared with the PRRSV group, the relative expression levels of Bax protein in the zinc sulfate treatment group was significantly reduced (*p* < 0.05, *p* < 0.0001) (Figure 8J), while the relative expression levels of Bcl-2 protein was significantly increased (*p* < 0.01, *p* < 0.0001) (Figure 8K). The results showed that PRRSV infection can significantly increase the expression of caspase3, cleaved-caspase3, and Bax proteins in Marc-145 cells, and significantly decrease the expression of Bcl-2 protein. However, zinc sulfate treatment can significantly inhibit the expression of caspase3, cleaved-caspase3, and Bax proteins, and significantly promote the expression of Bcl-2 protein.

## 4 Discussion

Zinc ranks as the second most essential trace element in biological systems, serving as a cofactor for over 750 transcription factors and 200 antioxidant defense enzymes (22). It plays a vital structural and catalytic role in fundamental processes, including DNA synthesis, RNA transcription, and oxidative stress resistance (22). Beyond its structural and metabolic contributions, zinc exerts robust antiviral activity through multiple mechanisms, including direct suppression of viral replication, modulation of host immune responses, and regulation of inflammatory signaling (23). Empirical evidence demonstrated zinc’s efficacy against diverse viruses, for example, influenza virus, rhinovirus, HIV, HCV, and Dengue virus (24–28). Consistent with these observations, our work demonstrated that zinc sulfate significantly inhibited PRRSV replication, reducing the expression of the PRRSV N gene and N protein in Marc-145 cells. To furtherly elucidate the molecular mechanism of zinc sulfate-mediated PRRSV suppression, we conducted genome transcriptomic analysis. The results demonstrated that the target genes of zinc sulfate against PRRSV were significantly enriched via pro-inflammatory mediators, signal transduction regulators, and apoptosis regulators. Therefore, we hypothesized that zinc sulfate exerts its anti-PRRSV effects by modulating inflammatory responses and apoptosis. To substantiate the transcriptomic analyses, we systematically investigated the effect of zinc sulfate on pro-inflammatory cytokines and anti-inflammatory cytokine and apoptotic pathway protein in PRRSV infected cells. In addition to, considering the pivotal role of oxidative stress in viral replicaition (29–31), the oxidative stress biomarkers also were evaluated.

ROS have been implicated in the virus infection. Multiple viruses, including Zika virus, HSV, influenza virus, rhinovirus, and respiratory syncytial virus (RSV), have been shown to disrupt redox homeostasis, resulting in excessive ROS accumulation (32–35). Excessive ROS accumulation induces oxidative stress, which will be exploited by viruses to enhance viral replication and favor viral persistence and dissemination (29). Our research data indicated that PRRSV infection triggered a marked increase in intracellular ROS levels. A similar study was also reported by Yan et al. (31), demonstrating that PRRSV disrupted redox homeostasis by exacerbating oxidative damage while impairing endogenous antioxidant mechanisms. The accumulation of ROS after PRRSV infection subsequently induces cellular autophagy, which suppresses NLRP3 inflammasome activation, thereby antagonizing the host’s innate immune response and ultimately facilitating PRRSV replication (29). Notably, exogenous antioxidant treatment markedly attenuates the elevated viral titers induced by oxidative stress (30). Zinc has long been recognized for its potent antioxidative capacity (36). Our study demonstrated that zinc sulfate significantly alleviated ROS level induced PRRSV infection. Given the positive correlation between ROS accumulation and PRRSV replication, this finding indicates that zinc sulfate probably inhibits PRRSV proliferation by reducing infection-triggered ROS accumulation. However, zinc per se lacks redox activity. But it adeptly preserves systemic redox homeostasis through modulation of key enzyme and protein activity (37). This is exemplified by zinc’s role as an essential cofactor for the Cu/Zn-SOD metalloenzyme, where it stabilizes the enzyme’s tertiary structure, protects its active site, and enhances its catalytic efficiency. The enhanced SOD catalyzes the dismutation of superoxide radicals into molecular oxygen and H₂O₂, while CAT subsequently detoxifies the resulting hydrogen peroxide by converting it into water and oxygen, collectively mitigating cellular oxidative stress (38). This is well supported by our data, which show that zinc sulfate efficiently reverses the diminished activities of SOD and CAT triggered by PRRSV infection. Through this coordinated enzymatic cascade, SOD and CAT collectively execute their antioxidative functions to mitigate ROS (38). Besides, zinc sulfate probably exerts its antioxidative effects through the following mechanisms: zinc ions effectively induce metallothionein (MT) expression, a protein with potent scavenging activity against ROS and reactive nitrogen species (RNS) (10); zinc modulates the Keap1/Nrf2 signaling pathway, leading to the upregulation of key antioxidant genes, including NAD(P)H, NQO1, HMOX1, GCL, GSH, and SOD (39). However, this preliminary conclusion, largely drawn from existing literature, requires rigorous experimental validation to establish causal relationships.

Viral infections typically trigger inflammatory cascades, which are the first line of defense against the spread of viral infections. However, uncontrolled inflammatory cascades often culminate in substantial host tissue pathology (40). The findings revealed that PRRSV infection triggers an aberrant overproduction of pro-inflammatory cytokines including IL-6, IL-8, IL-1β, and TNF-*α*, which in turn facilitates PRRSV replication (41). Remarkably, our findings demonstrate that zinc sulfate effectively suppresses PRRSV-induced pro-inflammatory cytokines (IL-6, IL-8, TNF-α) while concomitantly augmenting anti-inflammatory IL-10 expression in Marc-145 cells, suggesting its inflammation-modulatory potential. Consistent with our findings, Zhang et al. reported that zinc dramatically inhibited PEDV-induced secretion of IL-1β, IL-6, and IL-8 (19). These findings collectively suggest that zinc possesses potent anti-inflammatory properties and bolsters host defense mechanisms against viral pathogens, including PRRSV and PEDV. However, definitive evidence establishing zinc as a genuine anti-inflammatory agent remains elusive. But only limited evidence has confirmed that zinc supplementation ameliorates clinical manifestations of inflammation-associated disorders (36). NF-κB has traditionally been regarded as a canonical proinflammatory signaling pathway, primarily due to its activation in response to inflammatory cytokines like IL-1, IL-6, and TNF-α, as well as its critical role in regulating the transcription of other proinflammatory mediators, including cytokines, chemokines, and adhesion molecules (42). In view of these considerations, we hypothesize that the anti-inflammatory effects of zinc sulfate probably associated with its modulation of NF-κB pathway activity, and accordingly examined expression changes of key molecules in NF-κB pathway. Contrary to expectations, our results show that zinc sulfate activates the NF-κB pathway in PRRSV-infected cells, while accompanying proinflammatory cytokines reducing. Notably, however, anti-inflammatory cytokine IL-10 expression was observed to increase. Yu et al. demonstrated that NF-κB activation induces IL-10 expression in PRRSV-infected PAMs (43). Furthermore, the upregulation of this anti-inflammatory cytokine IL-10 posesses potent inhibitory effect on proinflammatory cytokine, particularly TNF-α (44). Therefore, we propose a mechanistic pathway whereby zinc sulfate triggers NF-κB activation to upregulate IL-10, which in turn downregulates proinflammatory cytokine synthesis via negative feedback. Beyond its inflammatory roles, NF-κB also orchestrate a diverse spectrum of physiological and pathological processes including immune modulation, cellular survival, apoptosis, proliferation, and differentiation (45). Therefore, the precise mechanisms by which zinc sulfate-activated NF-κB promotes cell survival in PRRSV-infected cells to suppress PRRSV replication remain to be fully characterized, highlighting an importance for future investigation. Notably, although Marc-145 cells serve as a conventional model for PRRSV research (20, 29–31), PAMs offer superior physiological relevance, especially for authentic immune responses like cytokine production. While we observed pronounced anti-PRRSV effects via zinc-mediated inflammation modulation in Marc-145 systems, PAM-based validation is warranted to establish physiological significance in subsequent studies.

Apoptosis has been identified as a crucial antiviral defense mechanism that effectively restricts viral replication (46). However, this cellular process is subsequently hijacked to facilitate viral dissemination among host cells. The study demonstrated that PRRSV-induced apoptosis exhibited a dual-phase mechanism: inhibitory during early infection (24 and 36 h) (47), where it detrimentally impacts viral replication, but progressively transitions to a proviral role at later stages (48 h and after 48 h), ultimately promoting viral proliferation (48). Similarly, our data demonstrates that the apoptosis is initiated by PRRSV infection at 48 h. Notably, zinc sulfate significantly inhibits apoptosis triggered by PRRSV infection at 72 h. This observation indicates that zinc sulfate inhibits PRRSV dissemination through apoptosis suppression. To investigate the mechanism underlying zinc sulfate-mediated inhibition of apoptosis, key proteins within the mitochondrial intrinsic apoptotic pathway were quantitatively analyzed. Our data reveal that zinc sulfate efficiently antagonizes PRRSV-mediated dysregulation of apoptotic effectors– suppressing caspase-3 and cleaved-caspase-3 activation, attenuating Bax upregulation, and restoring Bcl-2 expression. These findings are consistent with Srivastava et al. (49), who showed that zinc effectively suppresses influenza virus-induced apoptosis by inhibiting Caspase-3 activation and cleavage, thereby reducing viral replication. Mechanistically, zinc sulfate probably disrupts key effector proteins of the intrinsic mitochondrial pathway at initial apoptotic stages, leading a pronounced apoptosis decline in late-stage phase, consequently achieving PRRSV suppression. Additionally, zinc acts as a modulator of caspases, which exhibit zinc-dependent proteolytic activity. In the extrinsic apoptosis pathway, zinc chelates the catalytic cysteine, inducing steric hindrance that disrupts substrate-binding pocket occupancy (50, 51). In the intrinsic pathway, zinc coordinates the nucleophilic cysteine of caspase-9, preventing apoptosome assembly (50, 51). This dual inhibition abrogates proteolytic cascades, reducing apoptosis execution.

This study demonstrates that zinc sulfate exerts significant inhibitory effects on PRRSV replication through the modulation of oxidative stress, inflammatory responses, and apoptosis at the cellular level. These findings not only establish a critical foundation for elucidating the antiviral mechanisms of zinc against PRRSV but also offer a compelling theoretical framework for its potential clinical application in antiviral therapy. Nevertheless, the antiviral efficacy of zinc against PRRSV in animal models remains to be further validated, with the primary limitation being the scarcity of robust clinical data confirming its therapeutic potential against PRRSV. It is widely recognized in swine production that zinc is routinely employed as a feed additive to improve growth performance in pigs (52). In accordance with regulations established by China’s Ministry of Agriculture and Rural Affairs, the maximum allowable zinc supplementation in weaned piglet feed is permitted to reach 1,600 mg/kg (calculated as elemental zinc) during the initial two weeks post-weaning, while the upper threshold for other production phases is restricted to 110 mg/kg. But in actual production environments, no substantial antiviral effect of zinc against PRRSV has been documented. This phenomenon probably results from inadequate zinc bioavailability in swine, preventing the attainment of serum zinc concentrations sufficient to combat PRRSV infection effectively. Notably, zinc bioavailability varies significantly depending on the zinc source (52). The study measured the apparent total tract digestibility (ATTD) of zinc in weaned piglets and found that ZnSO₄ exhibited an ATTD of 8.7%, while ZnO showed only 3.42% (53). At a supplementation level of 100 mg kg^−1^, neither inorganic zinc (e.g., ZnO) nor organic zinc could maintain the baseline serum zinc level observed at weaning (852 μg L^−1^) (53). All groups exhibited declining serum zinc concentrations—for instance, the ZnO group decreased from 795 μg L^−1^ to 656 μg L^−1^ (53). Similarly, conventional dietary supplementation levels (50–100 mg kg^−1^) merely sustain serum zinc concentrations at 7.4 μmol L^−1^ (481 μg L^−1^) (54), which substantially lowers the therapeutically effective threshold required for cellular-level antiviral activity. Consequently, despite dietary zinc supplementation, the poor bioavailability prevents porcine serum zinc concentrations from attaining the therapeutic threshold necessary for effective PRRSV inhibition. Therefore, elevating serum zinc concentration should be a critical consideration in animal model validation studies of zinc’s anti-PRRSV efficacy.

The intracellular zinc pool maintains concentrations spanning tens to hundreds of micromolar; however, the bioavailable fraction remains constrained through tight protein coordination and sequestration in organelles and vesicles (55). Thus, cytosolic free zinc ion remains at minimal levels (in the picomolar range) (55). Such homeostasis is meticulously orchestrated by specialized transporter families: the ZnT (SLC30) proteins mediate zinc efflux to attenuate cytosolic concentrations, while the ZIP (SLC39) family facilitates zinc influx from both extracellular and subcellular compartments to replenish cytosolic pools (56). But excessive zinc supplementation triggers endocytosis of ZIP transporters on the cell membrane and promotes degradation of both ZIP proteins and their mRNAs (57). This regulatory mechanism restricts further zinc uptake, ultimately reducing zinc bioavailability. Notably, in cell culture, zinc ionophores, such as hinokitol (HK), pyrrolidine dithiocarbamate (PDTC), and pyrithione (PT), can stimulate zinc ion uptake within minutes (28). This implies that the combination of zinc ionophores with zinc sulfate significantly elevates intracellular zinc ion concentrations, concomitant with improving the bioavailability of zinc. And this will be necessary to evaluate the animal’s potential clinical applicability of zinc sulfate against PRRSV. Presently, zinc sulfate remains merely a provisional candidate for PRRSV therapeutic development.

## 5 Conclusion

**Figure 9:**
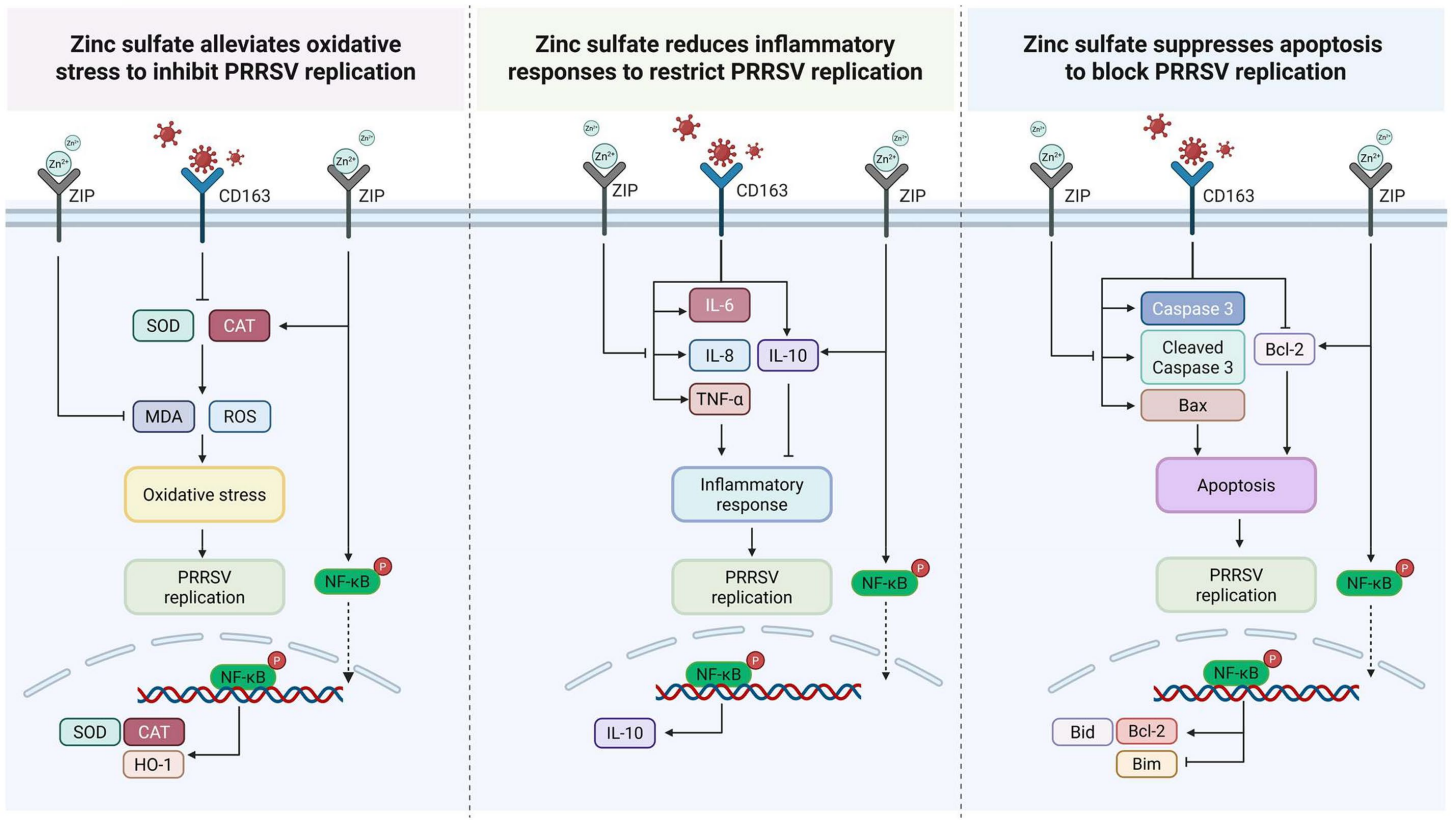
The potential mechanism of zinc sulfate against PRRSV: attenuation of oxidative stress, inflammatory responses, and apoptosis.

In conclusion, our findings indicate that zinc sulfate exerts antiviral effects against PRRSV by targeting vital genes associated with oxidative stress, inflammatory reactions, and apoptosis. Transcriptomic analysis identified these pathways as pivotal to the host response, and subsequent experimental validation confirmed that zinc sulfate alleviates PRRSV-induced oxidative damage, reduces inflammatory responses, and inhibits apoptosis, illustrating its potential as a therapeutic agent for PRRSV infection. A comprehensive elucidation of the underlying mechanism has been meticulously rendered and presented in Figure 9, which provides a detailed schematic representation of the following: zinc sulfate alleviates oxidative stress, reduces inflammatory responses, and suppresses apoptosis to block PRRSV replication.

## Data Availability

The datasets presented in this study are deposited in a publicly accessible online repository. The names of the repository/repositories and accession number(s) can be found below: https://www.ncbi.nlm.nih.gov/bioproject/PRJNA1309744.

## Footnotes

^1^ https://www.genecards.org

^2^ https://www.bioinformatics.com.cn
